# POSS-Based Polymers

**DOI:** 10.3390/polym11101727

**Published:** 2019-10-22

**Authors:** Joseph D. Lichtenhan, Krzysztof Pielichowski, Ignazio Blanco

**Affiliations:** 1 Hybrid Plastics Inc., Hattiesburg, MS 39401, USA; lichtenhan@hybridplastics.com; 2 Department of Chemistry and Technology of Polymers, Cracow University of Technology, Ul. Warszawska 24, 31-155 Kraków, Poland; kpielich@pk.edu.pl; 3 Department of Civil Engineering and Architecture and INSTM UdR, University of Catania, V.le A. Doria 6, 95125 Catania, Italy

The combination of functional polymers with inorganic nanostructured compounds has become a major area of research and technological development owing to the remarkable properties and multifunctionalities deriving from their nano and hybrid structures. In this context, polyhedral oligomeric silsesquioxanes (POSSs) have increasing importance and a dominant position with respect to the reinforcement of polymeric materials. Although POSSs were first described in 1946 by Scott, these materials, however, were not immediately successful if we consider that, starting from 1946 and up to 1995, we only find 85 manuscripts in the literature regarding POSSs. This means that less than two papers per year were published over 50 years. Since 1995, we observe an exponential growth of scientific manuscripts concerning POSSs. It has changed from an annual average of 20 manuscripts for the period 1995–2000 to an annual average of about 400 manuscripts, with an increase of 2800%. The introduction of POSSs inorganic nanostructures into polymers gives rise to polymer nanostructured materials (PNMs) with interesting mechanical and physical properties, thus representing a radical alternative to the traditional filled polymers or polymer compositions.

Polyhedral oligomeric silsesquioxanes, with Si vertices interconnected by –O– linkages, form three-dimensional nanometer size cage structures with substituents attached to silicon atoms. These substituents may contain reactive groups, such as hydroxyl or isocyanate. A combination of a rigid inorganic nanocore with organic vertex groups makes POSS molecules useful hybrid building blocks that can be chemically incorporated in the polymer matrix by copolymerization, grafting or reactive blending, or physically mixed by solvent casting or polymer processing by using, for example, the extrusion technique. Depending on the number and kind of functional groups attached to the POSS cage, silsesquioxane moieties can be chemically built into a polymer structure as a side group of the main chain or terminating end-group, as a main chain fragment, or as a macromolecular network knot.

Various routes of chemical decoration of POSS molecules with organic substituents offer new perspectives for synthesis of novel organic–inorganic hybrid materials with desirable—and often still unknown—properties. Interest in POSS-containing polymer composites and hybrid materials has been growing in the recent years as they show improved mechanical performance, thermo(oxidative) stability, and surface durability. Well-defined structure, non-toxicity, and enhanced biocompatibility, as well as biostability against oxidation, hydrolysis, and enzymatic attack under in vitro and in vivo conditions make functionalized silsesquioxanes desirable nanofillers in the biomedical field as implants and scaffolds. Other application areas include membranes, high-performance adhesives, flame retardants, aerogels, optical sensors, and shape-memory materials, to name a few. On the other hand, challenges with polyhedral oligomeric silsesquioxanes’ tendency to agglomerate are still to be addressed during synthesis and engineering of nanostructured polymer-based composites made thereof.

This special issue, which consists of 18 articles, including two review articles, written by research groups of experts in the field, considers recent research on novel POSS-based polymeric materials.

Firstly, it was highlighted that, compared with the other most commonly used fillers, POSSs possess the advantage of being molecules, thus allowing the combination of their nano-sized cage structures, which have dimensions that are similar to those of most polymer segments, and production of a particular and exclusive chemical composition. These characteristics linked with their hybrid (inorganic–organic) nature allow researchers to modify POSS according to particular needs or original ideas, before incorporating them into polymers [1].

Liu et al. synthesized a novel organic–inorganic hybrid containing allyl benzoxazine and polyhedral oligomeric silsesquioxane (POSS) to be used for preparing epoxy resin composites in order to verify the effect of POSS on the thermal stability and flame retardancy of a prepared material. Improvement in thermal stability and flame retardancy was observed when the amount of POSS reached 10% or more, thus demonstrating that POSS nanoparticles can effectively protect the combustion of internal polymers [2].

Wei and co-workers, by using the graft-from method, prepared a series of heptaphenyl siloxane trisilanol/polyhedral oligomeric silsesquioxane (T_7_-POSS) modified by polyols to be used for obtaining polyurethane nanocomposites at different POSS contents. The results showed that the polyol-terminated POSS particles overcame the nano-agglomeration effect and evenly dispersed in the polymeric matrix. The damping factor of resultant nanocomposites increased from 0.90 to 1.16, while the glass transition temperature decreased from 15.8 to 9.4 °C when POSS contents increased from 0 to 9.75 wt%. They attributed the improvement of the damping properties of the composites to the friction-related losses occurring in the interface region between the nanoparticles and the matrix [3].

POSS-derived Si@C anode material was prepared by Bai et al. with the copolymerization of octavinyl-polyhedral oligomeric silsesquioxane and styrene, for use in a lithium ion battery. The initial discharge capacity of the battery based on the as-obtained Si@C material Si reached 1500 mAh g^−1^. After 550 charge–discharge cycles, a high capacity of 1430 mAh g^−1^ was maintained. By using combined XRD, XPS, and TEM analysis they showed the high potential of the novel electrode material and provided insight into the dynamic features of the material during battery cycling, which will be useful for the future design of high-performance electrode material [4].

Chen and collaborator used cationic octa-ammonium polyhedral oligomeric silsesquioxane (Oa-POSS) particles to improve the performance of traditional sodium alginate (SA) hydrogels that meet a greater application demand in the biomedical field. The characterization of the gels demonstrated that their properties depend on the content of both the uniformly dispersed Oa-POSS and poly(*N*-isopropyl acrylamide) (PNIPA) network directly. Furthermore, they observed that the gels with a hydrophilic PNIPA network exhibited better swelling ability and remarkable temperature responsiveness, and their volume phase transition temperature can be adjusted by altering the content of Oa-POSS. The deswelling rate of gels increases gradually with the increase of POSS content due to the hydrophobic Si–O skeleton of POSS [5].

By monitoring the polymerization kinetics by Photo-Differential Scanning Calorimetry (DSC) and photorheology, Marcinkowska et al. investigated the effect of monomethacryloxy-heptaisobutyl POSS (1M-POSS) on the process. They determined that at low concentrations the modifiers with a bulky substituent increase the molecular mobility by increasing the free volume fraction, which leads to an acceleration of the termination and slows the polymerization. At higher concentrations, they retard molecular motions due to the “anchor effect” that suppresses the termination, leading to acceleration of the polymerization. Thus, they considered the possibility of anchoring a monomer with a long substituent around the POSS cage, which further enhances propagation [6].

Song et al. synthesized a series of hybrid thermoplastic polyurethanes from bi-functional polyhedral oligomeric silsesquioxane and polycaprolactone using 1,6-hexamethylene diisocyanate as a coupling agent for testing their thermo-mechanical properties. They observed an increase in decomposition temperature and glass transition temperature when compared with pristine polyurethane, and an improvement of both storage modulus (G′) and loss modulus (G″), showing the possibility to further adjust these parameters by varying POSS content in the copolymer. In addition, the synthesized POSS polyurethanes demonstrated a remarkable effect in toughening commercial polyesters, indicating a simple yet useful strategy in developing high-performance polyester for advanced biomedical applications [7].

The modification of polyurethane foams with aminopropyl isobutyl-POSS (APIB-POSS) and aminoethylaminopropylisobutyl-POSS (AEAPIB-POSS) to enhance their mechanical and thermal properties was the object of the study of Członka and her collaborators. The results showed that the morphology of modified foams is significantly affected by the filler typology and content, which resulted in inhomogeneous, irregular, large-cell shapes, and further affected the physical and mechanical properties of resulting materials. The best results were obtained for the polyurethane modified with 0.5 wt% of APIB-POSS, showing greater compression strength, better flexural strength, and lower water absorption [8].

Aiming to bridge the gap between small-molecule surfactants and amphiphilic block copolymers Qian and coworkers designed and synthesized single-tailed giant surfactants carrying hydrophobic poly(ε-caprolactone) (PCL) as the tail and a hydrophilic cage-like polyhedral oligomeric silsesquioxane (POSS) nanoparticle as the head. To endow the POSS head with adjustable polarity and functionality, three kinds of hydrophilic groups, including hydroxyl groups, carboxylic acids, and amine groups, were installed to the periphery of the POSS molecule by a high-efficiency thiol-ene “click” reaction. The full characterization demonstrated that these giant surfactants can form nanospheres with different sizes in aqueous solution [9].

Li et al. synthesized, by a one-step grafting reaction, a hybrid flame retardant copolymer starting from methacryloisobutyl polyhedral oligomeric silsesquioxane (POSSMA), reactive glycidyl methacrylate (GMA), bis-9,10-dihydro-9-oxa-10-phosphaphenanthrene-10-oxide methacrylate (bisDOPOMA), and derivative functionalized graphene oxide (GO). They showed a remarkable enhancement of the composites’ thermal properties by adding the graphene oxide hybrid flame retardant (GO-MD-MP). Furthermore, they observed an increase in the limiting oxygen index as well as the mechanical strength of the epoxy resin [10].

Wang et al. prepared blends of cyanate ester and phthalonitrile–polyhedral oligomeric silsesquioxane and studied their cure behavior by means of thermal and rheology experiments. The obtained copolymers showed high chemical reactivity, low viscosity, and good thermal stability. In addition, an increase in glass-transition temperature of the blends, compared to cyanate ester resin, was recorded, making them suitable for preparing carbon-fiber-reinforced composite materials via a winding process and a prepreg lay-up process with a molding technique [11].

The effect of preparation method, POSS content, and type, on the morphology, thermal, mechanical, and surface properties of poly(ε-caprolactone)/POSS derivatives nanocomposites (PCL/POSS) were studied by Cobos et al. Morphological analysis evidenced that amino-POSS with a longer alkyl chain exhibited a better degree of dispersion independent of preparation method, reducing the formation of POSS crystalline aggregates. They also showed how the incorporation of POSS derivatives into the PCL matrix improved thermal stability and enhanced the surface hydrophobicity of PCL [12].

Ueda and collaborators designed and prepared, by the casting method, dual-functionalized polyhedral oligomeric silsesquioxane (POSS) derivatives, which have seven fluorinated alkanes and a single acrylate ester on the silica cube, to be used as a filler for lowering the refractive index and improving the thermomechanical properties of poly(methyl methacrylate) (PMMA). They observed a large lowering of the refractive index. Moreover, the degradation temperatures and the storage moduli of the obtained films were greatly elevated by loading the POSS fillers [13].

Niemczyk and coworkers were engaged in the evaluation of a novel series of siloxane-silsesquioxane resins as possible flame retardants in polypropylene (PP) materials. Their results revealed that the functionalized resins formed a continuous ceramic layer on the material surface during its combustion, which improved both thermal stability and flame retardancy of the PP materials. This beneficial effect was observed especially when small amounts of siloxane-silsesquioxane resin were applied [14].

Li et al. prepared two different models of hybrid ionic liquids (ILs) based on polyhedral oligomeric silsesquioxanes (POSSs), showing excellent thermal stability and low glass transition temperatures. They then focused their attention on the high sensitivity of these products for detecting nitroaromatic compounds, highlighting their great potential for the detection of explosives [15]. 

Lin and collaborators prepared and characterized mesoporous molecular sieves by using rice husk as a silicon source, thus also solving environmental pollution problems to avoid its burning as garbage, in addition to the desired field of application. Structural characterization showed evenly distributed and hexangular mesoporous structures. An increase in pore size was obtained, thus leading to an increase in ammoni–-nitrogen adsorption capacity [16].

Finally, this special issue hosts two reviews. The first one, by Dudziec et al., highlights the significant number of papers on the design and development of POSS-based organic optoelectronic as well as photoluminescent (PL) materials. In view of the scientific literature abounding with numerous examples of their application (i.e., as Organic Light Emitting Diodes (OLEDs)), the aim of the review was to present efficient synthetic pathways leading to the formation of nanocomposite materials based on silsesquioxane systems that contain organic chromophores of a complex nature. A summary of stoichiometric and predominantly catalytic methods for these silsesquioxane-based systems to be applied in the construction of photoactive materials or their precursors was given [17].

The preparation of hybrid nanocomposite materials derived from polyhedral oligomeric silsesquioxane (POSS) nanoparticles and polyimide (PI) was the subject of the second review presented by Mohamed and Kuo. The two researchers discuss the various methods used to insert POSS nanoparticles into PI matrices, through covalent chemical bonding and physical blending, as well as the influence of the POSS units on the physical properties of PIs [18].
